# Human-Centered Explainable Artificial Intelligence: Automotive Occupational Health Protection Profiles in Prevention Musculoskeletal Symptoms

**DOI:** 10.3390/ijerph19159552

**Published:** 2022-08-03

**Authors:** Nafiseh Mollaei, Carlos Fujao, Luis Silva, Joao Rodrigues, Catia Cepeda, Hugo Gamboa

**Affiliations:** 1 LIBPhys, Physics Department, Faculty of Sciences and Technology, Nova University of Lisbon, 2825-149 Caparica, Portugal; lmd.silva@fct.unl.pt (L.S.); jmd.rodrigues@fct.unl.pt (J.R.); c.cepeda@campus.fct.unl.pt (C.C.); hgamboa@fct.unl.pt (H.G.); 2 Volkswagen Autoeuropa, Industrial Engineering and Lean Management, Quinta da Marquesa, 2954-024 Quinta do Anjo, Portugal; carlos.fujao@volkswagen.pt

**Keywords:** natural language processing, functional work ability, occupational health protection profiles, explainable AI (XAI), musculoskeletal symptoms

## Abstract

In automotive and industrial settings, occupational physicians are responsible for monitoring workers’ health protection profiles. Workers’ Functional Work Ability (FWA) status is used to create Occupational Health Protection Profiles (OHPP). This is a novel longitudinal study in comparison with previous research that has predominantly relied on the causality and explainability of human-understandable models for industrial technical teams like ergonomists. The application of artificial intelligence can support the decision-making to go from a worker’s Functional Work Ability to explanations by integrating explainability into medical (restriction) and support in contexts of individual, work-related, and organizational risk conditions. A sample of 7857 for the prognosis part of OHPP based on Functional Work Ability in the Portuguese language in the automotive industry was taken from 2019 to 2021. The most suitable regression models to predict the next medical appointment for the workers’ body parts protection were the models based on CatBoost regression, with an RMSLE of 0.84 and 1.23 weeks (mean error), respectively. CatBoost algorithm is also used to predict the next body part severity of OHPP. This information can help our understanding of potential risk factors for OHPP and identify warning signs of the early stages of musculoskeletal symptoms and work-related absenteeism.

## 1. Introduction

Staff health protection can be a competitive advantage for industries because it promotes (1) job safety and importance [1], (2) minimizes medical expenses [2], while saving absence costs by preventing injuries, and (3) reduces industry expenses and the forced absence of skilled, specialized, and experienced company workers, leading to overall industry improvement and worker’s satisfaction [3].

Industries have distinctive policies to protect their employees’ health. When employees are exposed to risk factors that cause work-related musculoskeletal diseases, technical teams are responsible for protecting their health in industrial environments. Sharing the findings from their risk assessments and supporting the decision-making process on the match between job demands in each workplace and employees’ remaining work capacity is one possible contribution [4].

Bernardes et al. [5] and Assunção et al. [6,7] declared that the process of industrial ergonomics has a significant role in the decision-making process in this automotive industry where this study took place, as they are also responsible for risk assessment for occupational biomechanical overload risk factors. Given the worker’s health condition, the matching method entails/implies Occupational Physicians (OP) assigning each worker an authorized exposure to occupational risk factors. To put it another way, Occupational Health Protection Profiles (OHPPs) are created by OPs without the need for clinical data. These profiles are written in Portuguese and report on constraints in both physical and organizational working circumstances. In the worker’s OHPPs, the OPs record the Functional Work Ability (FWA). The definition of FWA is functionally changed to “capacity” (or, in other words, work ability), as this data is directly reported on the Occupational Health Protection Profiles. The “prediction” focused on predicting the changes in FWA given, for example, the following factors: 1. Work-related factors 2. Personal Factors 3. Organizational factors. In the automotive industry, where the study took place, the ergonomics team periodically screened the workplace conditions as well as the occupational health department’s screening of health conditions, and this practice may have influenced the employees’ health maintenance. To look for any potential imbalances between job demands and FWA, it is critical to examine the work capacity profiles of employees exposed to high physical demand activities, such as those in the automotive sector. Data on the variation in physical ability among employees who perform physically demanding jobs would be helpful to support occupational and health policies to encourage healthy workplaces at all ages. This study offered a way to recognize the predicted factors linked to the functional deterioration in the automotive industry. In this case, it records one or more comments about the worker’s FWA based on the worker’s health status in the system so that the technical teams can do the necessary planning for the continuation of the worker’s job demands. When each worker goes to the medical department, OP start by providing the worker’s standard information, namely the personnel number, the worker’s area, etc., are registered in the company’s system. The workers also go through a measure of their Functional Work Ability (FWA) in the worker’s OHPP. In this case, it records one or more comments about the worker’s FWA based on the worker’s health status in the system so that the technical teams can do the necessary planning for the continuation of the worker’s job demands without aggravating worker’s health status. Each comment refers to a different body part, and occupational physicians assign one of two labels, “MN-Must Not Use” (MN) or “SN-Should Not Use”, (SN) based on the degree of risk to that body part(s). The MN label indicates more severe conditions than the SN. In addition, the body parts whose ability changed in a worker’s FWA status in OHPPs are targeted.

**Figure 1:**
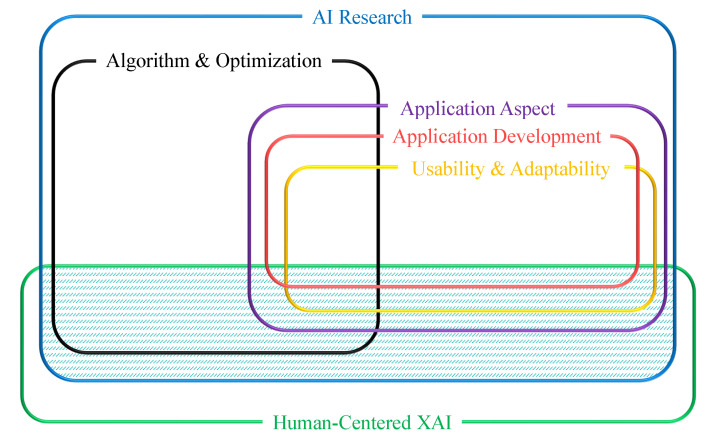
Human-Centered design (marked with dashed lines) research lays over a broad spectrum, as shown here. The intersection of AI Research and Human-Centered Design is the domain we identify as HCXAI [10].

Researchers have been involved in the treatment of musculoskeletal symptoms, while others have focused on preventing them (see as an e.g., [8]). This assessment is a sort of HCXAI that connects humans and Artificial Intelligence (AI) (Figure 1). However, even in this case, if we train AI models statically on the previous data, by changing the environmental conditions and manpower, other features may be involved in the damage or new damage may be created by that efficiency [9]. It also reduces the quality of the trained model. As a result, technical teams such as OP and ergonomists can interact with AI to maintain the current models by updating them, adding new data to the models, and providing feedback on the AI prediction results. This example, which is a sort of Human-Centered Explainable Artificial Intelligence (HCXAI), can fill the gap between humans and AI by transforming academic and static AI models into functional and dynamic models.

This association between humans (health) and AI (introduced here as a HCXAI) could be used to assess the organizations’ capacity to retain their manpower in their working settings and to cope with work-related absenteeism. Previously, researchers attempted to solve the capacity of organizations to retain their manpower in their working settings and how to cope with work-related absenteeism problems with different health variables by combining different data mining techniques or merging different models. For example, [11] used decision tree algorithms for classification purposes in dividing the absence time (hours) into four categories for classification analysis: “hours”, “days”, “weeks”, and “not absent”. This category can be based on less than 5 h or more than 5 h [12]. For the regression scenario, different neural networks such as Long Short-Term Memory (LSTM) were used in [13,14]. Overall, it can help the company’s costs and prevent decreased workers’ FWA of trained, specialized, and experienced company employees, which leads to the improvement of the productivity [3].

For technical teams to validate why and how a certain choice was taken, an AI strategy should also include not just precise algorithms, but also interpretable and explainable approaches (see various fields e.g., [15,16]). There is a shortage of research that has used eXplainable AI (XAI) [17,18] to predict medical appointments, its severity, and how to prevent work-related musculoskeletal disorders by means of identifying the body parts with the lowest FWA. Regarding [14], there is a gap in previous research to determine the best ways of comparing and evaluating the performance of various regression models when employing specific features. The advances of XAI research in Natural Language Processing (NLP) are evidenced by the main categories of explanations in various methods for arriving at and visualizing explanations (Local vs. Global, Self-Explaining vs. Post-Hoc [19,20]). Danilevsky et al. detailed operations and explainability techniques currently available for generating explanations of model predictions, in the hopes of serving as a resource for developers interested in building explainable NLP models [21].

There is a growing demand for AI frameworks that are reliable, transparent, interpretable, and explainable to humans. The Python SHappley Additive exPlanations (SHAP) package (https://github.com/slundberg/shap, accessed on 31 May 2022) allows us to calculate SHAP values for a selected model and it has already been broadly used [22]. Recently, a new class charting tool, known as decision diagrams, has been added to the SHAP package. This instrument provides a detailed view of the inner workings of a model, which means that it allows us to understand how models make decisions. This is an opportunity to propose a comprehensive study in how SHAP features impact approaches [23] in order to identify the most significant characteristics.

Besides, HCXAI improves prediction performance and ensure that the findings are trustworthy and reliable. The current study focuses on using interpretable and information-based machine learning algorithms to make such predictions. The proposed method can help industries undertake assessments, design strategies, and, finally, prevent musculoskeletal symptoms and its consequences.

As a proposed method in the area of HCXAI studies, the following questions will be addressed in particular:

- What are the most effective ways to explain supervised classifiers?
- Is it possible to transpose HCXAI algorithms that include visual explanations to emphasize correlations between samples and features?
- What are the most important prerequisites for the automatic generation of supervised classifier decision explanations?
- What do technical teams need to know about an intelligent system to trust its decisions or feel at ease working with one?
- Is it possible for the intelligent system to convey this knowledge to its technical teams in a meaningful and understandable way?

The creation of a new information measure known as objective-based entropy, which takes into account the ordinal nature of the target, is one of the research’s significant contributions. We also aim for interpretable models as decision-making aids in technical teams. The HCXAI model is useful for assessing and predicting musculoskeletal symptoms by body parts because it combines interpretable modeling with a metric that takes occupational profile data into account.

The paper’s major technical contribution is a novel case-based reasoning approach for assisting domain experts in preventing musculoskeletal symptoms that yield work-related absenteeism. We present [24,25,26] Gradient Boosting Decision Trees (GBDT) that consider the output model in explanation, which is different from past work in this field. We compared the GBDT algorithms to get better error evaluation in this research. To the best of our knowledge, this is the first study to use XAI based on the medical history of worker profiles to predict the next medical appointments in two scenarios: (i) predict the next medical appointment according to weeks; and (ii) predict next FWA body parts (severity) by using SHAP.

We have empirically demonstrated that our approach can be useful for alert demanding jobs and is recognized as such by domain teams. As a result, it can contribute by modeling an indicator to assess the capacity of organizations to retain their manpower in their working settings and how to cope with work-related absenteeism. The findings could also be used to prevent work-related musculoskeletal disorders because they identify the body parts with the lowest FWA. More specifically, our objectives and contributions are as follows:

- Investigating Machine Learning (ML)-based techniques and elicit essential features OHPPs by selecting the most relevant parameters collected in real working environments.
- Finding the most suitable prognosis protocols in real working as the second objective, by comparing ML-based enhanced worker’s health profile models.
- Causality and explainability of these ML algorithms refer to a human-understandable model since it is measured in terms of efficacy, efficiency, satisfaction related to causal understanding, and transparency for industrial technical teams like Ergonomists.

The rest of this article is structured in the following way: Section 2 reviews the previous related work. Section 3 describes the data collection method and the proposed approach of this research in detail. Section 4 provides experiments and results, and Section 5 provides conclusions and suggestions for future work.

## 2. Related Work

Nowadays, AI plays a crucial role in many fields, including monitoring health status. In addition, with the electronic registration records about reasons for absenteeism, such as vacations, marriages, births, deaths, address changes, and health problems, analysis of this data can be beneficial for data science researchers. Using this AI method also helps in decision-making concerning the prevention, diagnosis, and treatment of diseases. For example, Kakhki et al. [8] predicted the extent of the body part(s) damaged in accidents occurring at grain handling facilities using decision trees and naïve Bayes algorithms. In this way, the frequency of such incidents can be reduced, and medical indemnity payments are prevented. In another study, Kim et al. [27] used NLP techniques to extract keywords from pathology reports in electronic health records. They trained the KEA [28] and WINGNUS [29] deep learning models through supervised learning and then evaluated them on labeled and unlabeled data.

Any research trying to address the black-box challenge for AI is referred to as XAI. Despite the benefits of intelligent systems, the XAI research effort raises concerns about giving them too much power without the ability to explain the decision-making process that lies beneath such complex systems to domain teams in terms and in a format that they can understand [30]. This not only assists in comprehending specific decisions made by such systems, but it also encourages them to develop more human-like solutions, as well as encouraging further research and understanding of the brain as a natural information processing phenomena. Furthermore, intelligent computers are still unable to interpret abstract information or real-world knowledge until it is transformed into a format that the algorithm can understand. XAI attempts to assist the technical team [31] in identifying why a machine decision was made and whether or not it is reliable. As a result, XAI is necessarily a paradigm for bridging machine intelligence and their intelligence, to enable and broaden human adoption of AI systems. In this sense, XAI stands for “AI for human”.

Because worker’s productivity is one of the most relevant factors in organizations, XAI become a higher attraction for technical teams in different fields. In other words, these studies used supervised learning to present XAI [18,32] as a promising result in many areas of decision-making [33]. For example, explainable recommendations [34] enable system designers in comprehending why a recommender system proposes a specific product to its electronic medical records. It helps in the improvement of a recommender XAI system’s performance as well as the clarity of medical decision-making for patients. Then, by analyzing online health, they identified the relationship between the disease and the medicine [35]. In another case, detecting musculoskeletal disorders using these AI approaches is difficult because it cannot be accurate enough for Complementary and Integrative Health (CIH) for pain treatment as an alternative to opioid medications [36]. For research purposes, datasets that are used for musculoskeletal disorders such as musculoskeletal symptom detection are developed, but the recorded ones cannot be constant and dynamic. Because it is a sensation caused by certain medical disorders, it is difficult to predict, as it can be with other medical conditions (like, ref. [6]).

The productivity of employees and workers in organizations is such an important health issue that it has caused technical teams in various companies to pay attention to it. Extracting meaningful reasons from OHPPs leads to discovering new knowledge that technical teams can use to advance companies towards greater development and higher productivity. Although previous research that used XAI from staff companies’ data has provided useful information to organization executives, none has addressed the SHAP based *shapley* values as relevance scores regarding the decision-making process. Particularly, this research proposes a small but informative way to evaluate the potential of the visual explanations produced by our original method to be adopted in a real environment. Results show the best regression in terms of two different scenarios.

## 3. Methodology

By regulation, occupational health surveillance is complementary [37]. Based on the health policy of this automotive industry, at least every two years, each worker should be scheduled for a health appointment by the OP or whenever changes in workers’ work-ability are identified. OHPPs are assigned to workers to ensure that the work-ability of workers and the task they can perform is taken into account when deciding the tasks that worker has to perform. Each OHPPs is recorded in a digital file and presents the dimensions considered to establish the desired OHPPs, reproducing several data fields. NLP techniques were used to read the data fields to extract the keywords in an Excel database. After finding associations between body parts, regression algorithms were used in the prognosis scenario to predict the next medical appointment and the body parts’ severity determinants. In this section, our proposed method for predicting the next medical appointment and the body parts’ ability affected from records with data related to workers with changes in FWA. This process has been done in two phases:

- In the first phase (Section 3.1), the required data was collected and prepared.
- In the second phase (Section 3.2), the knowledge contained in the final data was extracted and analyzed using the regression method.

The results from the second phase will be relevant not only to understand what is the next medical appointment for each worker (week) but also for predicting body parts based on different categories, namely gender, seniority, and different automotive areas. Hence, by these results, technical teams can figure out how different job demands are influencing changes in workers’ work-ability, but also which working conditions improvements should be considered.

### 3.1. Data Description

In the automotive industry, decreased FWA are a significant concern as they can result in work absence. Whenever they are identified by the OP, an OHPPs is assigned in order to ensure that workers continue to attend to work. Data from three-year OHPPs assigned to workers in the Portuguese automotive industry were used in this study. Each OHPPs has 12 data fields describing either organizational (n = 6), individual (n = 2), work-related parameters (n = 4), and time (n = 2). The most relevant data fields for our study are the ones reported as “work-related”, meaning changes in FWA data. They are presented by a three-layer structure: the first layer states the level of protection; the second layer refers to the deviation on FWA, which will always be reported to one of the fourteen (14) body parts: neck, trunk, shoulder (L (Left) and R (Right)), elbow (L and R), wrist (L and R), hand fingers (L and R), knee (L and R), foot (L and R), and the third layer reports to the additional information labeled as “comments”. The database designed for this study uses 3 of the 12 data fields: one from the organizational cluster (area), one from an individual cluster (employee number), and one from a work-related cluster (OHPPs). To properly extract the text from this last cluster, an NLP protocol was designed.

As OHPPs are written by the OPs in text format, several deviations were found between different OPs, and between different periods for the same OP. Minor deviations, like non-word symbols, extra spaces, misspellings, and incorrect use of capital letters, were found. Additionally, major ones were also identified, like the first layer, which addressed the criteria “MN-Must Not use” (MN) and “SN-Should Not use” (SN) being written at the end of the sentence instead of in its beginning. We used NLP techniques to process each cleaned sentence and extract the keywords for the “body part” and “risk label” assigned to it by the (OP)s. Whenever an OHP does not explicitly describe the body part under protection but instead assigns processes, e.g., “manual material handling” or “vibration tools”, no body parts were reported in the database. That decision was made because it was established that only OPs can assign it. Finally, we stored the obtained information in the form of tabular data structures. Each row of the final table is a record of a worker, and each of the 14 columns in this table represents a body part. Each item in the table indicates the type of risk for a worker’s body part (MN or SN). As an example, Table 1 shows two rows of the final tabular of such structured data.

As stated before, MN protection categories are addressed by OPs to ensure the highest level of health protection possible, leading to a procedure whose final result is the identification of jobs that match with workers’ FWA. Regarding occupational exposure management, protections classified as MN lead to the absence of the correspondent risk factor. Therefore, theoretically, the dose has to be equal to zero regardless of the risk exposure dimension: duration, frequency, and intensity. Although the automotive industry’s working systems are complying more and more with ergonomic design criteria, working conditions are still far from meeting zero dose exposure. Therefor, MN protections might represent the intended protection and not the worker’s remaining FWA. Since the risk exposure dimensions are used to match job demands with FWA, it might be an advantage to review the occupational health protection assignment procedure. Instead of qualitative expressions targeted at intended health protection, quantitative data related to the risk exposure dimensions should be preferred. We know that occupational technicians in this industry, in addition to discovering the FWA, also pay attention to the restrictions on the body parts MN or SN. In the Table 1, we divided each column of body parts into two columns: one for the MN label and one for the SN label. For example, the Shoulder_L column has been split into the $Shoulder_{L-MN}$ and $Shoulder_{L-SN}$ columns. As a result, we obtained a table with 28 columns and cells that contained the numbers 0 and 1.

### 3.2. Data Pre-Processing

**Figure 2:**
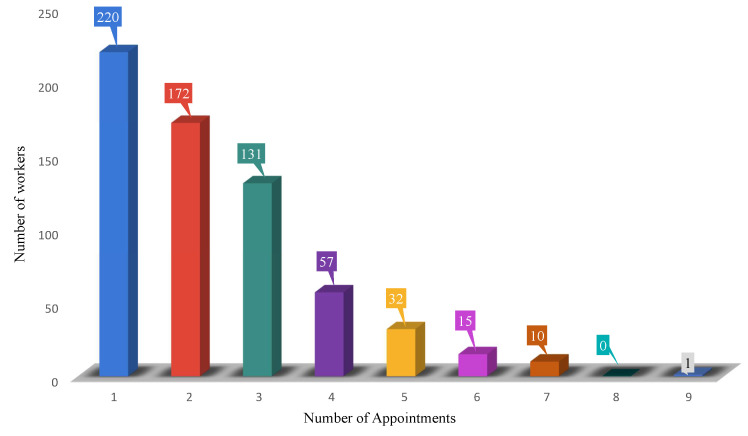
Medical appointment distribution based on the number of workers.

We used the Natural Language Toolkit (NLTK) (https://pypi.org/project/nltk/, accessed on 31 May 2022) to clean up the OHPPs. In this section, 7857 OHPPs were based on the FWA status of workers. The preprocessing procedure was based on the description in Section 3.1 was targeted. So, 1514 records were achieved after cleaning the data. According to the quantity of medical visits, workers’ records are grouped in Figure 2 so that the size of each category gets smaller as the number of appointments increases. In addition to classifying the records of employees’ medical appointments according to the number of appointments, we also divided the records into groups of 418 workers who only have medical appointments for each worker.

These records were for 638 workers, 23.3% were women and 77.7% were men, respectively. Therefore, males have a higher rate of medical appointments. Table 2 describes the seniority portion of workers according to male and female. Additionally, mean and Standard Deviation (SD) are presented based on male and female criterion.

Among these OHPPs, the histogram is illustrated below as a statistical summary of 638 workers based on medical appointments. Figure 2 shows workers’ record grouping based on the number of medical appointments in such a way that the size of each category decreases with the number of appointments. In our data, there is no worker with 8 medical appointments.

**Figure 3:**
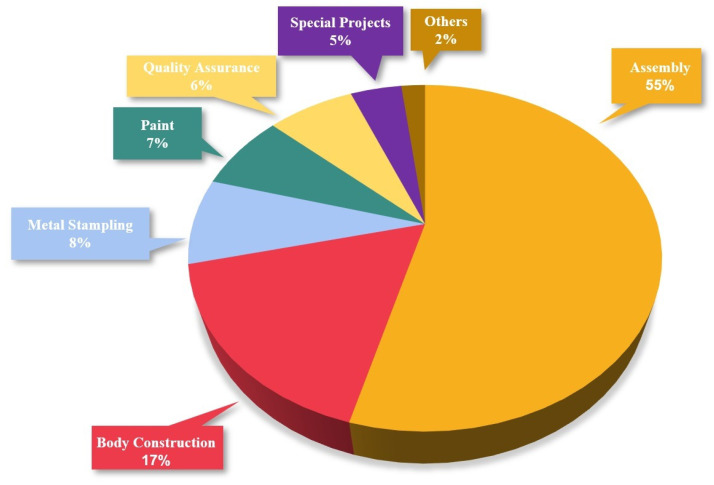
Distribution of workstations in OHPP records between 2019 to 2021 (>7 k records).

In addition to categorizing workers’ medical appointment records (1514 records for 638 workers) by the number of appointments, we also categorized these records by removing workers with a single medical appointment (418 workers). Such categorizing is presented in Figure 3, in which there is an unambiguous relationship between working in different areas and the number of workers there. As mentioned before, other areas such as Special Cars, Product Management and Planning, Logistics, Plant Manager, and Finance contain less than 2% of the records from which reliable knowledge can not be extracted. Therefore, we have not considered these areas for this research. It should be noted that our data was obtained directly from an automotive company, and was collected by the company itself based on workers’ medical appointments.

### 3.3. Proposed Method

**Figure 4:**
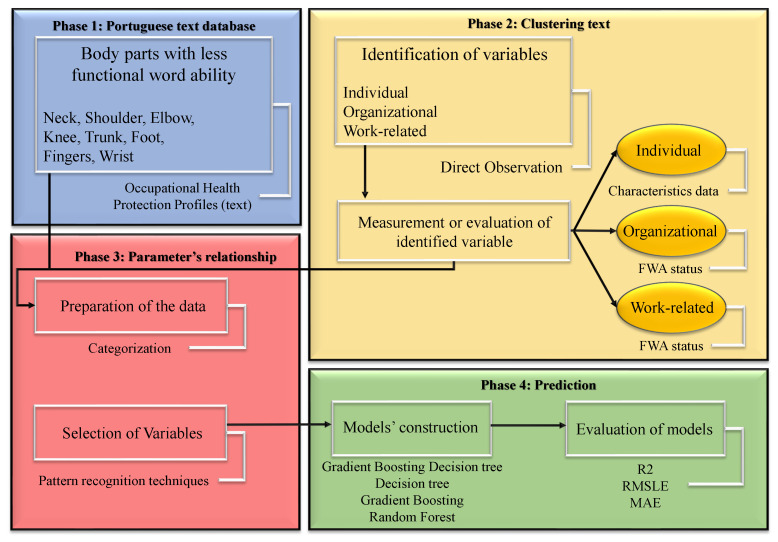
Phases of the method process used in this investigation.

Empirical research methods are a class of research methods in which empirical observations or data are collected to test a theory [38]. In the present study, a quantitative method serves to test this theory of whether utilizing worker´s FWA parameters can improve the performance of ML approaches in OHPPs. This section describes the four phases of the methodology applied in this research work (Figure 4). The first prognosis phase centers on a GBDT [39,40,41,42,43] and study of preceding research work on AI. This phase has three stages: studying the academic occupational profiles such as workers or patients, studying the AI algorithms, and studying the AI evaluation methods. Regarding the principal research objective that deals with improving AI algorithms in academic medical investigation, the most popular academic Machine Learning-based Natural Language Processing (ML-NLP) [44] was first analyzed comprehensively. The next stage of the first phase contains a detailed description of the most important physical keyboards in FWA statues (described in Section 3.1). The second section is devoted to providing a point out of the OHPPs text. About the database described in Section 1, workers’ FWA status is used to create OHPPs. In other words, workers’ health protection departed from three factors that are criteria for analyzing AI algorithms. These metrics fall into three main groups based on what exactly they belong to: (1) work-related parameters; (2) individual variables and (3) organizational criteria. HCXAI, supporting decision-making, aims to go from worker’s FWA to explanations by integrating explainability into Medical Restrictions (MRs) AI and supporting OP in decision context: prognosis of individual, work-related and organizational parameters. The selection of relevant predictor variables chose using an expert-driven methodology in the automotive industry. This kind of selection of relevant predictor variables was crucial during the construction of regression models because not all the variables contained information directly associated with FWA associated with musculoskeletal disorders and some acted as significant variables that only impaired the quality of the results. The first phase is related to protections based on eight body regions in Portuguese texts.

Physical loading (external) from outside the body produces physiological effects within the body. Individual capacity refers to the traits that define how well body tissues can withstand external stimulus and how well they respond physiologically to it. If the biomechanical forces are too great, direct tissue damage will occur. The capacity is determined by [45]:

- Body type and size: Strong and large people can withstand heavier loads than weak and little people.
- Gender: Women’s maximal muscle strength is around two-thirds that of men, regardless of body size [46,47].
- Age: Muscle strength increases during adolescence but begins to decline before the age of 30. This loss begins slowly, but it accelerates with age, reaching an average of 8–16 percent every decade after 50 years of age (in this study age is excluded) [46,47].
- General health: Several disorders can weaken tissues and cause injuries.
- Skills: Skilled persons can manipulate their bodies and external forces to keep biomechanical forces within the body at a minimum. Unskilled people are more likely to be involved in situations that result in accidental injuries (e.g., when losing their balance). Seniority and function are two factors in this category.

The fourth section introduces the criteria for analyzing regression algorithms. The prediction of the next medical appointment and next FWA body part(s) was treated as a regression problem, where the expected outcome was a continuous value. Several regression methods were considered, according to the type of data collected and the final objective of the study, namely Decision Tree (DT) regression, Random Forest (RF) regression, GBDT, Gradient Boosting (GB), and GBDT. The regression methods were compared to identify the one with the highest predictive level. These metrics fall into two main groups based on what exactly they measure: measuring the rating prediction R Square (R2), usage prediction error RMSLE (RMSLE), Mean Absolute Error (MAE), and SHAP evaluation in XAI.

Regarding the prediction of the next medical appointment, as Figure 4 shows, this study devised four main phases, briefly divided as:

- phase 1: **Portuguese text database** the database is updated in the automotive industry weekly. This database, entitled “OHPP”, includes the FWA status of workers according to their body parts.
- phase 2: **clusteringtext** using NLP to extract individual, organizational, and work-related factors by identification of appropriate variables. There is other information in the OHPP database that can give us other organizational and work-related parameters, such as the place where workers work, workers’ functions,…
- phase 3: **parameter’s relationship** using pattern recognition for finding relationship.FWA body parts of workers have multi-side associations, based on the area where workers work.
- phase 4: **prediction** model’s construction with regressor models such as GBDT, DT, GB, and RF by error evaluation such as R2, RMSLE, and MAE. These predictions are related to two scenarios: the next medical appointment of workers and the next FWA body parts (severity).

### 3.4. Work Ability Index Scoring

Work Ability Index (WAI) was measured as a subjective comparison of current workability to a person’s self-identified lifetime. The WAI was created at the Finnish Institute of Occupational Health and tested using clinical data [48]. The WAI is a tool that is used in clinical occupational health care and research in many countries (it has been translated into 26 languages). The index is calculated using responses to a series of questions about work demands, worker health, and resource availability. Scores range from 0 (unable to work) to 10 (maximum workability), with excellent (scoring 10) being the highest, good (score 9), moderate (score 8) being the next highest, and poor (scores 0–7) being the lowest. Work capability is divided into two categories: good (8–10) and poor (0–7). In this study, due to the body parts of workers assigned to medical appointments, the range of (0–7) took into account. Therefore, according to Table 3, each body part is weighted based on the level of protection of Must Not_used (MN) and Should Not_used (SN). For example, one of the workers had the medical restrictions below:

- Must not perform tasks that involve movements above both shoulder line
- Should not perform tasks that involve performing flexion/rotation movements of the trunk
- Must not perform tasks that imply performing tasks using tools with associated vibration
- Should not perform tasks that involve performing left and right wrist rotation movements
- Must not perform tasks that involve performing tasks that require force with application point on the fingers of both hands

Categorization for each medical restriction starts with SN = 1, MN = 0.5. Scoring MN Use: We had two MN-Must Not Use. Therefore, the scoring point for 2 MN-Must Not Use is *2*. We had to divide this score between two sentences and their body injured parts. In this case, 0.5 goes to shoulder_R, shoulder_L and also finger_L, and finger_R. We have two SN-Should Not Use, which are a score of *5* to injured body parts. This score is also divided by two, being given a score of *2.5* for the first and second sentences. We have to score the trunk as *1.66* and wrist_R and wrist_L as *0.8*. Finally, the scoring would be given by:

Table 4 provided, based on Neupane’s (2011) multi-site pain and work ability among an industrial population, whether the number of musculoskeletal pain sites predicts future poor work ability.

The highlighted goal of this study is to investigate the performance class categories [49] in GBDT regression methods used in the next medical appointment with protection body parts prediction and to determine the explainability feature selection method that can be used with the SHAP methodology to improve visualization prediction accuracy. Nevertheless, making ML regressions more explainable is far from being an easy task. For example, following the tree pathway from a decision tree, might be used to “explain” the predictions made by decision trees. However, a large number of nodes could make this procedure awkward and related explanations nearly incomprehensible to humans. Furthermore, integrating ML models into technical teams, especially OP support systems, is a difficult task in and of itself, because these models must be based on uncertain, imbalanced, heterogeneous, and noisy datasets, which contain other features but are often insufficient to allow precise modeling of occupational profiles.

Each type of regression model consists of simply two steps. In the first step, data normalization is performed using a standard scaler, and the second step is where the actual regression algorithm occurs. We began the data pre-processing and performed the regression modeling next. The regression modeling was selected, including RF, H_2_O, GB, CatBoost, XGboost, LightGBM, DT. The output of each produced model was logged during each iteration, based on the features chosen and the data mining techniques used, and the findings were presented when the entire process was finished. To train regression models, we considered 80% of the dataset, which included 332 records related to workers’ FWA status, as training data and the rest (84 records) as test data. As the R2 metric can give a comprehensive view of the model performance and capabilities, we also consider it as a 10-fold cross-validation metric for performance comparisons. The dataset contains numerical features indicating the scoring extent of fourteen workers’ body parts (Table 4). It also includes encoded categorical features related to the worker such as their zone, organizational unit, function, work schedule, and seniority. Therefore, for the first scenario, which is predicting the next medical appointment, all the variables that we mentioned in the dataset are inputs and the data for the medical appointment is the output. Besides, for the second scenario, which is to predict the next body parts’ severity, FWA body parts are our output.

## 4. Experiments and Discussion

In this section, we present the results of implementing the proposed approach. As we mentioned in Section 3.3, for evaluating the capability of all regression models based on different metrics, we considered 80% of our dataset, including 332 records for model training, and the rest as the test data.

### 4.1. ML Regression Models Result for Prediction of Next Medical Appointment

As a first objective, we are about to predict the time of the next medical appointment based on the worker’s FWA. In order to create the target label out of the next medical appointment in date, we considered the time interval from a default time source in terms of the number of weeks. The existence of outliers might cause the error term to expand to a very high value in the case of RMSLE [50]. The outliers in RMSLE, on the other hand, are dramatically scaled-down, thereby nullifying their influence. To put it another way, the RMSLE is resistant to the impact of outliers. The RMSLE error is not impacted by the inclusion of the outlier. The relative error between the expected and actual values is referred to as the RMSLE. The penalty for underestimating the actual variable in RMSLE is likewise higher than the penalty for overestimating it. Since our data are such that a significant number of workers have only medical records and the number of workers with more records of a medical visit has been lower, selecting a criterion such as RMSLE seems logical which can show the ability of the model better. As the results of the Table 5 show, the CatBoost regressor, which is based on GBDT, is able to produce impressive outcomes. The primary principle behind boosting is to progressively integrate multiple weak models (models that perform marginally better than chance) to generate a strong competitive prediction model using greedy search. This characteristic of the regressor explains why it performed so well with our data. This method has been able to obtain the best RMSLE, which indicates its excellent fit on the train data, which allows them to learn from them and perform a good estimation on the test data. Additionally, the R2 score of this method over different cross-validation folds shows its superiority over other methods.

#### 4.1.1. Visual Performance Analysis of ML Regression Models

**Figure 5:**
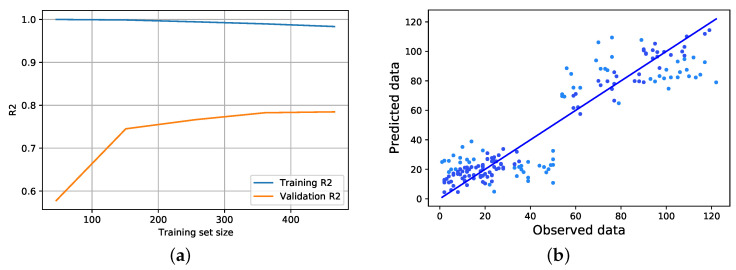
CatBoost Regressor (**a**) learning curve and (**b**) true vs predicted data plots.

Learning curves show how a learning algorithm’s models perform in terms of generalization as a function of the size of the training set. For analyzing the different designed models’ learning process more visually, we considered 80% of the data for training them and the remaining 20% as the test data for their learning trend comparison. As Figure 5a illustrates, the CatBoost regression model starts from the very beginning with its most overfitted learning process, which at this stage does not provide a good result on the validation OHPPs data. In the continuation of the process, by increasing the size of the data, the model almost maintains its sensitivity to noisy or outlier data, which causes its relative overfitting. However, over time, as the process goes through, it can achieve more than the R2 score on the validation set occurs in the range of 0 to 150 records of the training set and then slows down as it reaches the 400 records of the training set. The CatBoost regressor Figure 5b, which had the best performance in terms of regression results, is also one of the best examples in terms of prediction-vs-observed plot.

#### 4.1.2. Model Analysis Using SHAP Value

As ML models become more widely employed, it is becoming increasingly critical to comprehend their performance. The traditional ML measures such as MAE and R2 score, among others, do not provide deep insight into the model’s performance. We can have regression ML models with a high R2 score, but it is really a discovery of features that should not be utilized for prediction.

SHAP employs a game theory method to explain model predictions. We concentrate on how to utilize SHAP to examine the performance of the regression model in this domain. It begins with a base value for prediction based on past knowledge and then tests other features of the data one by one to see how the addition of that information affects the base value before making the final prediction. In other words, the ML model’s predictions for each instance can be reproduced as the sum of these SHAP values plus a fixed base value, resulting in:

(1) f(x)=basevalue+sum(SHAPvalues)

The mean of the target variable is used as the base value in regression models. It also considers the sequence in which features are introduced as well as their interactions, allowing us to better understand model performance. It captures SHAP values throughout this procedure, which will be used to visualize and explain predictions afterward. SHAP values give important insights into how the input factors are affecting the predictions of the ML model, both at the level of individual instances and throughout the population as a whole.

SHAP is a model-agnostic approach that can be applied to any ML algorithm, so the details of the modeling process are unimportant for this discussion. It can be a useful approach when models have suspiciously high prediction results arising from information leaks from the target variable to the feature set. The SHAP features a set of classes known as explainers that may help comprehend a variety of ML models. The following are some examples of helpful and frequent sorts of explainers [22]:

- Generalized Additive Models are explained using **AdditiveExplainer**.
- For linear models accessible from sklearn (https://scikit-learn.org/, accessed on 31 May 2022), **LinearExplainer** is used. It may also take into consideration the link between features. The python SHAP package (https://github.com/slundberg/shap, accessed on 31 May 2022) is used for plotting common useful SHAP plots, which are presented for further visualized performance analysis in the following.
- **TreeExplainer** is a model that is built on a tree, such as a decision tree, a random forest, or gradient boosting.

We chose TreeExplainer for our analytic objective based on the descriptions of the specified explainers and soft hyperparameter tuning was used for Catboost Regressor as our selected model.

Additionally, to evaluate the aforementioned model, plots were made to show the predictions of the model in comparison to the real body part values, both in the training set and in the test set. In this way, the model achieves better efficiency in estimating the predicted values in the test routine, in which it is validated with a portion of the data set with which it had no contact in the training routine. Additionally, we observed that the distance between the predicted and actual values decreases considerably, obtaining an acceptable metric.

**Figure 6:**
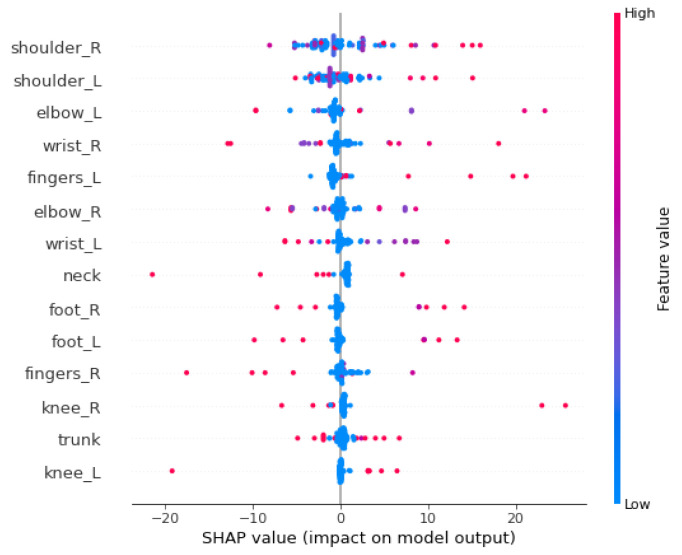
Summary plot per body parts for test data. The x-axis indicates the changing amount to the SHAP value by top effective factors. Such factors are arranged in descending order from top to bottom based on their impact on the y-axis.

Figure 6 illustrates the summary plot on the test data in which the contribution of the different body parts (Table 4) are shown on the y-axis. The higher the variables are placed on the y-axis, the more they influence the final model prediction. The magnitude of their contributions is represented by the colored line on the right from high (red) to low (blue). The x-axis represents the SHAP values of each of the variables by which the model predicts the number of weeks till the next medical appointment (weekly) for workers with physical problems.

The interpretation of the summary plot shows that the presence of the variable shoulder right (lower values visible in blue on the horizontal bar) implies a decrease in time of medical appointment (expressed on the scale at the bottom of the figure); conversely, high FWA in shoulder right (visible in red) is associated with an increase in the predicted length of time of week of medical appointment effected individuals per worker’s body part. It means that if the value of shoulder right is high then it increases the amount of functionality ability the worker has and takes less time for the next medical appointment. The same analysis can be applied to the rest of the variables. SHAP sets a model’s mean prediction (base value) and determines the relative contribution of each feature to the target’s divergence from the base. It has the ability to provide both local and global explanations.

Local explainability helps to understand the model’s decision-making process for a single particular sample, while in global explainability, the focus is on all of the records. In other words, global explainability performs across all predictions. We have used both types of explainability for the comprehensive analysis of our model, and their details are presented in detail as follows.

**Figure 7:**
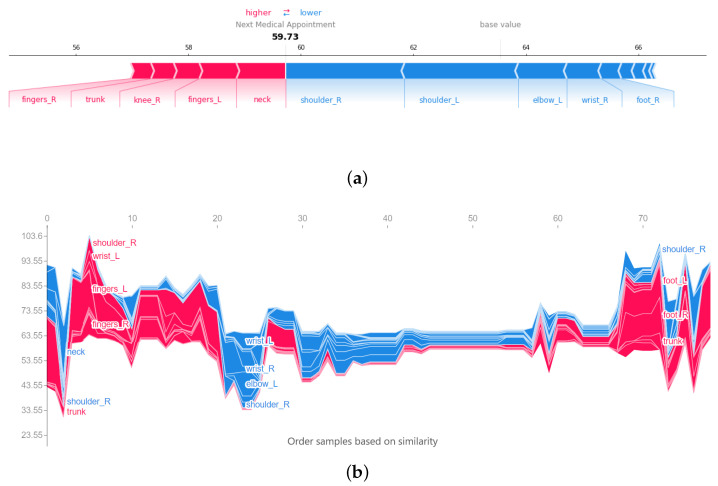
Force plot for (**a**) one record where the variables that increase the prediction of the model the most are shown in red and those that reduce the prediction of the model the most in blue. The numbers in the black line represent the number of weeks infected workers per body parts according to the base value of the training set (66) and the output value of the model’s prediction (59.73) for a sample record and (**b**) all records sorted by similarity. By a 90-degree rotation of the y-axis at a single point on the x-axis, the (**a**) sub-plot will be achieved.

**Local explanation**. The force plot of a particular random sample is presented in Figure 7 on which the method is explained. The force plot illustrates the output prediction and the contribution of each predictor which are retrieved based on SHAP value calculation on this sample. A similar pattern to the train data has shown by a force plot in Figure 7a. The force plot indicates the decreases and increases in the effect of the most influential body part factors on altering the base value (indicated on the horizontal bar with a value close to 64) to achieve the final predicted value (59.73).

Regarding the condition of this worker, the decrease in the function of the shoulders and left elbow has the greatest effect in reducing the time period until the next medical appointment. It seems that because the working conditions of these workers in the workplace require that you make the most of your hands and put pressure on them, these members are more involved and exposed to injury. This is while, although the increase in the function of the neck and fingers of his left hand has increased the time period until the next medical appointment, due to the lack of significant involvement of these organs, they have not made a significant impact.

**Global explanation**. Figure 7b is a global explanation of model prediction in which the 63.55 is the base value obtained using the SHAP value. We can observe that the condition of the neck and right shoulder functionality has resulted in a drastic decrease in the model output as the first falling trend of the plot. However, the status of their trunk had a negligible effect. As the second changing trend shows, for another group of workers, the condition of their fingers, right shoulder, and left wrist has delayed their need for medical attention as the model output. The last significant alteration to the model output happens at the end of the plot for another group of workers. In this way, the problem with their right shoulder’s functionality has resulted in a decrease in the time distance to the next medical appointment. However, the functional status of their feet and left knee could not help their health condition considerably.

**Figure 8:**
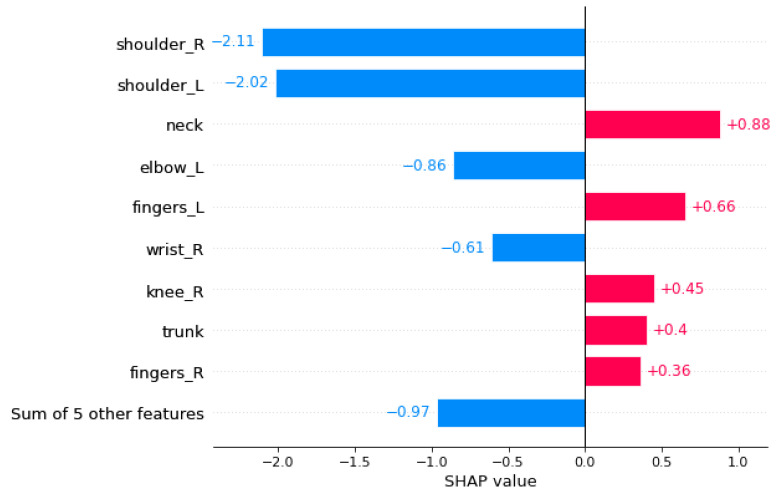
Bar plot for 5 sample records. On the y-axis, the most influential factors are arranged from top to bottom based on their contributions to the model output. The x-axis indicates their changing amount to the SHAP value.

Figure 8 also indicated that the health state of upper body parts such as shoulders, neck, left fingers, and left elbow are more contributing to the model output, as they are typically engaged in working by hand.

The decision plot depicts the decision process by applying the SHAP values of individual features one by one to the expected value in order to build a line chart with the projected value.

**Figure 9:**
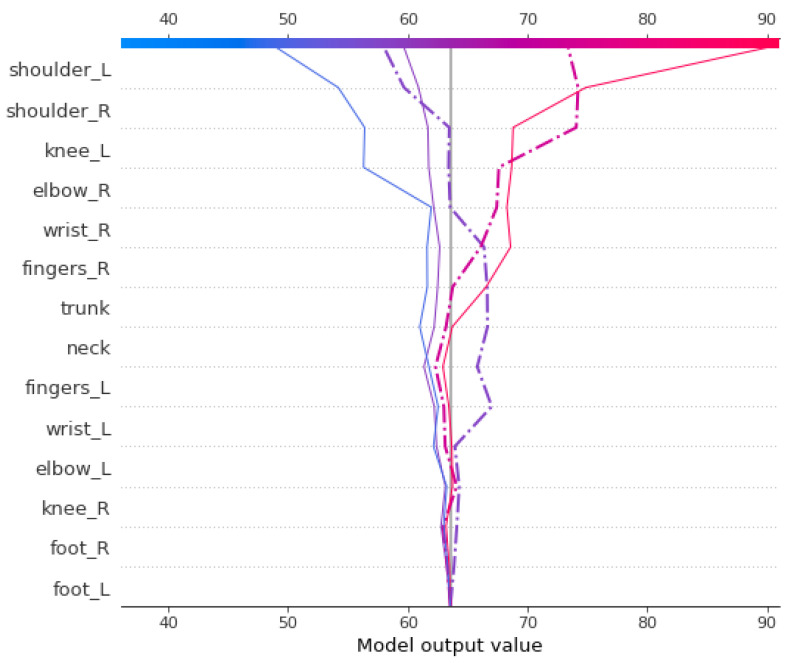
Decision plot for 5 sample records in which the prediction made by the model for each particular observation is shown.

The similar plot in Figure 9 also shows the decreasing trend of the effect of features on the final estimate of the model, so that in the highlighted records, the performance status of the left and right shoulders is the most influential factor, and its functionality affects the period until the next visit to the OP remarkably. At the lower level of feature importance, the influence of factors such as knees, elbows, wrists, and fingers of the right hand are mentioned as less influential factors.

SHAP analysis gives companies the possibility to investigate the conditions of the working environment by checking the amount of pressure on the workers’ organs in different sections. As a result, they will be able to reduce the work pressure and fatigue of the workers by making adjustments in the production and tools, and in this way, they will preserve more human resources, which is itself precious capital.

### 4.2. Regression Models Result in Next FWA Body Parts

For our second research objective, we predict the amount of severity to each part of the body at the last medical appointment, based on the OP’s FWA statues in OHPPs. For this purpose, we considered the physical constraints of the last medical report as a measure of the estimation. In this section, the results of body part severity related to different areas of the body based on different models are shown. The first evaluation criterion was RMSLE. The lower the value of this criterion, the better the model fits the data and the less error. The second evaluation criterion (R2) is a statistical measure of how close the data are to the fitted regression line. Therefore. the higher it is, the better the model performs, and the better the regression line fits the data. In the tables, the value of R2 is also reported for the validation data. We choose the R2 measure as a 10-fold cross-validation statistic for performance comparisons, since it provides a thorough perspective of the model’s performance and capabilities. Table 6 shows the results of the workers in health protection in this period for the left shoulder. According to the RMSLE criterion, the CatBoost performed better than the other five models. It also has the highest value of R2 on the validation data, as its boosting schemes help to reduce overfitting and improve the quality of the model.

Some of the main features of the CatBoost model are that it can achieve great results without hyperparameter tuning, there is no need a preprocessing to be performed on categorical features and it is computationally fast. Moreover, because it is less prone to overfitting, it can achieve decent accuracy.

According to the R2 criterion, the Light Gradient Boosting Machine (LightGBM) model performed better than the other 5 models. LightGBM has better accuracy than any other boosting algorithm, it produces complex trees by following a leaf-wise split approach rather than a level-wise approach which is the main factor in achieving higher accuracy. However, it can sometimes lead to overfitting, which can be avoided by setting the max_depth parameter.

Considering the MAE metric, simpler tree-based models, such as the DT and RF, could achieve better results, but all models’ MAE values are acceptable.

Table 7 shows the results of the workers that had health protection in this period for the right shoulder. According to the RMSLE criterion, the CatBoost performed better than the other five models. It also has the highest value of R2.

According to the R2 criterion, the LightGBM model performed better than the other 5 models on validation. Since it is based on DT algorithms, it splits the tree leaf-wise with the best fit, whereas other boosting algorithms split the tree depth-wise or level-wise rather than leaf-wise. Therefore, when growing on the same leaf in LightGBM, the leaf-wise algorithm can reduce more loss than the level-wise algorithm and hence results in much better accuracy, which can rarely be achieved by any of the existing boosting algorithms. It uses a novel technique of Gradient-based One-Side Sampling (GOSS) to filter out the data instances for finding a split value, while XGBoost uses a pre-sorted and Histogram-based algorithm for computing the best split. All regression models have achieved considerably low MAE values on this dataset, which shows their acceptable capability.

Table 8 shows the results of the workers having health protection for the left elbow. The number of workers with body injuries in the left elbow area is small in the data set. Data for this category has been scarce, so simpler models will work better on this data.

According to the RMSLE criterion, the XGBRegressor model performed better than the other 5 models. XGBRegressor can work well in small to medium datasets, and handle missing data with its in-build features [51]. XGBoost uses DTs as base learners; combining many weak learners to make a strong learner. As a result, it is referred to as an ensemble learning method, since it uses the output of many models in the final prediction.

According to the R2 criterion, the DT model performed better than the other 5 models. It is one of the quickest ways to identify relationships between variables and the most significant variable. DTs are not largely influenced by outliers or missing values, and they can handle both numerical and categorical variables. Since it is a non-parametric method, it has no assumptions about space distributions and classifier structure.

CatBoost has the highest value of R2 on the validation data because its structure reduces overfitting and improves the generalization of the model.

Although DT and RF models could achieve the best MAE error, they could not become the superior models in comparison with other models.

Similarly, Table 9 shows the results of the workers having health protection for the right elbow. According to the RMSLE criterion, the CatBoost performed better than the other five models. It also has the highest value of R2 on the validation data.

The primitive learning approach of the first two models seems to make them able to get the lower MAE results, although it could not help them to achieve the best overall results among all models.

According to the R2 criterion, the RF performed better than the other five models. The random forest algorithm provides a higher level of accuracy in predicting outcomes than the decision tree algorithm because, in a RF regression, each tree produces a specific prediction. The mean prediction of the individual trees is the output of the regression.

The CatBoost performed better than the other five models in terms of RMSLE and CV-R2 on trunk FWA dataset (Table 10). The LightGBM performed better than the other five models in terms of R2 and MAE. A high R2 score indicates the generalization point of view. Models are performing well.

#### 4.2.1. First Scenario Learning Curves

**Figure 10:**
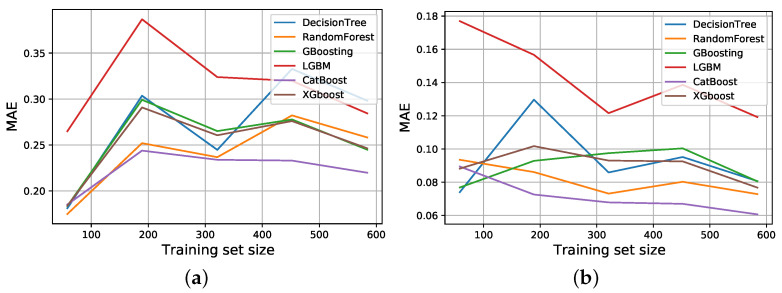
Learning curves of models for (**a**) right elbow and (**b**) left fingers.

Figure 10a shows the learning curve of models for the right elbow injury prediction. It indicates when the data size reaches a certain level that is sufficient for the model learning process and prevents the occurrence of overfitting, the error rate of most models such as CatBoost, XGbooslreaningt, RF, and GB, decreases with increasing data size. However, as the fitting process is expected to become more suitable, the DT models fitting on training sets with larger sizes seem not to follow the same pattern. The LightGBM model showed the worst fitting pattern on all possible training sizes, with considerable difference in MAE in comparison to other models.

The estimation of possible left-hand finger injuries for the models is shown in Figure 10b. As the figure illustrates, although models could finally achieve good performance at the maximum size, increasing the training set size does not seem to show a clear correlation with the final model error, and in both cases, the CatBoost model performance is remarkably good. Other models have shown similar downward performance, except for LightGBM, which can indicate that this model is more of a fast computational model than a comprehensive one.

#### 4.2.2. CatBoost Algorithm in Prediction Next Body Part Severity Based on Gender, Seniority, and Areas

A pie chart shows the relationships of parts to the whole for a variable. In this section, we examined the percentage of FWA in different areas of the body in different categories using pie charts. Pie charts show the predicted body part severity of workers who have worked in the automotive industry for a certain category. The OP and ergonomist can assign the exposure to these workers based on this category such as FWA body parts in men/women. For example, trunk and shoulder right are the factors that OP and ergonomist should consider for gender category, respectively. Quality assurance is the area that has the most impact on the trunk, and it is important that OP and ergonomists are aware that the gender of workers may have more issues in this area.

**Figure 11:**
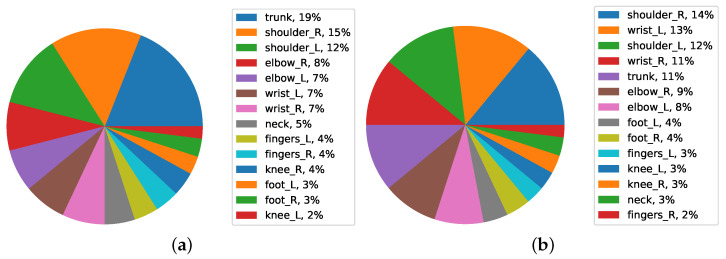
Average FWA of body parts in men (**a**) and (**b**) women data.

Figure 11b shows that men have the most job demand in the first level from the trunk, with 19%, and in the second place from the right shoulder, with 15%. The highest percentage of FWA in test data is from the trunk. The shoulders are on the second level, and the rest of the body has a small percentage of the FWA. In general, it can be said that both men and women have body parts FWA in the area of the right shoulder, with similar values. However, men have the least FWA body from the trunk, with 19%.

Considering the same plot for women, they have the least FWA in the first level from the right shoulder with 14/% and the second level from the left wrist with 13/%. After the left shoulder and right wrist with 12/% in the third level, the rest of the body has a small percentage of severity. There is evidence that exposure to occupational risk factors is likely to affect FWA in these body parts.

If the results are also analyzed based on the medical history of the worker, a very good view of the FWA loss can happen in relation to the work experience of the worker.

**Figure 12:**
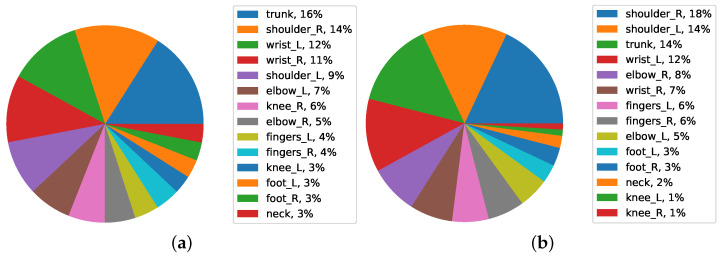
Average FWA of body parts in seniority of (**a**) 1–10 and (**b**) 10–20 data.

The Figure 12a determines which area has suffered the most in workers. According to the analysis, the highest percentage of FWA loss in workers in the first level was the trunk, with 16%, and in the second level, the right shoulder, with 14% and wrists, with 12% in the third level. The rest of the body has a small percentage of job demand.

Considering workers with 10 to 20 years of work experience (Figure 12b), the highest percentage of FWA loss is related to their right shoulder, with 18%, followed by their left shoulder defects, with 14%, and their trunk, with 14% in the third level. The rest of the body has a small percentage of FWA loss.

**Figure 13:**
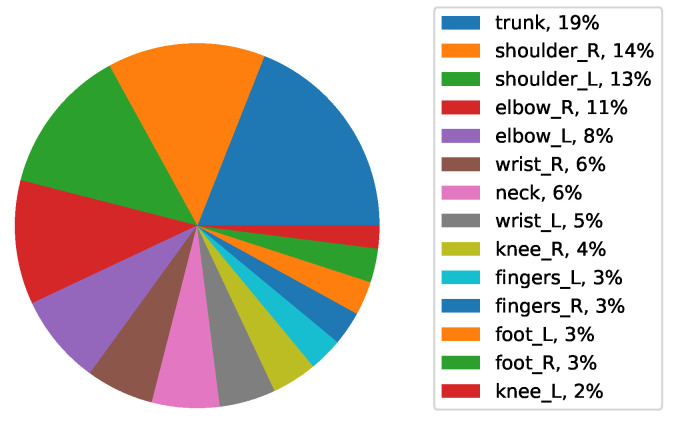
Average FWA of body parts in seniority over 20.

According to the analysis of workers with a work experience of more than 20 in Figure 13, the highest percentage of FWA loss in these workers is related to the trunk, with 19% followed by the right shoulder with 14% and the left shoulder with 13% in the third level, while the rest of the body parts have a small percentage of FWA loss.

The reason for the FWA loss in areas such as the right shoulder, which can be related to the fact that most of the workforce is right-handed, increases with increasing work experience. Trunk FWA loss is another problem that arises with experienced workers.

As FWA assessments based on a worker’s occupation can give a beneficial view of their job demand and workload. In this section, the extent of FWA loss based on the occupation of workers in categories such as Paint, Body construction, Assembly, Special Project, and Metal stamping, is examined.

**Figure 14:**
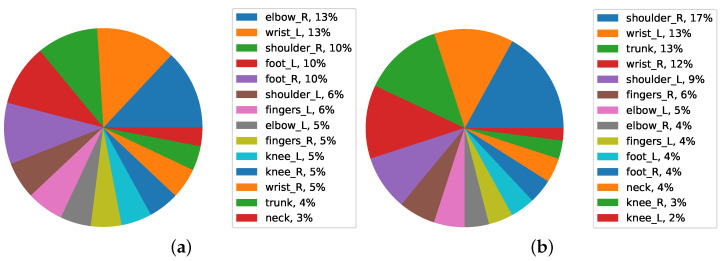
Average FWA of body parts in Special Projects for (**a**) and (**b**) Assembly data.

Figure 14 shows the pie chart for the special projects category. In the special projects, most of the body parts were related to the right elbow and the left wrist in the first level both with 13%. After the right shoulder, left foot, and right foot with 10% in the third level, the rest of the body has a small percentage of FWA. The “cause” was because the exposure to occupational risk factors had severe consequences on their work-ability as they were targeted with OHPPs at the highest protection level. Typically, the OHPP of workers belonging to “special projects” is the most challenging in terms of the match between remaining work ability and job demands. Whenever a match is not verified, a worker is not fit at all to work at the automotive factory, and therefore, the worker will be absent from work.

Regarding the Assembly data in the Assembly category, most of the data were related to the right shoulder in the first level with 17% and the left wrist and trunk both in the second level with 13% and right wrist with 12%. The data suggests that an assembly area can have, at the same time, the cause of the problem and the solution to it. There is evidence that exposure to occupational risk factors is likely to affect FWA in these body parts. It also means that the assembly has jobs that are compliant with OHPP in assigning the same body parts.

**Figure 15:**
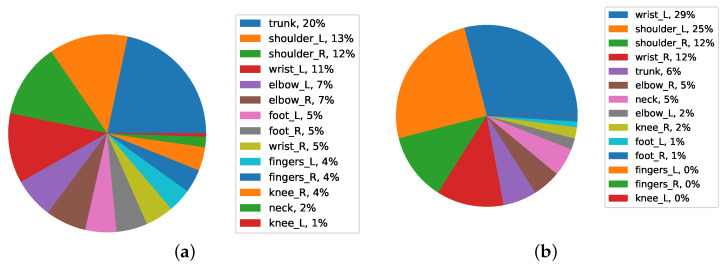
Average FWA of body parts in Body Construction for (**a**) and (**b**) Metal Stamping data.

Figure 15 shows the pie chart for the body construction category.

For the body construction category of test data, most of the worker’s health protection was related to the trunk in the first level (20%), and the left shoulder in the second level (13%). The highest rate of FWA was allocated with a different percentage related to the trunk. As the protection given is addressed to the highest level possible, it has, somehow, to be associated with exposure to working conditions.

The Metal Stamping category is shown on the pie chart in Figure 15b. Most of the health protection of workers was related to the left wrist in the first level (29%) and the left shoulder in the second level (25%). At the first level, the highest percentage is specialized in the left wrist. This analysis is practical for related decision-making because the workers from whom these results were reported were still working in this same production area. Having said that, it means that jobs matching the OHPPs were found in the Metal Stamping. The “solution” also applies because, under these circumstances, workers are not contributing to work-related absenteeism.

**Figure 16:**
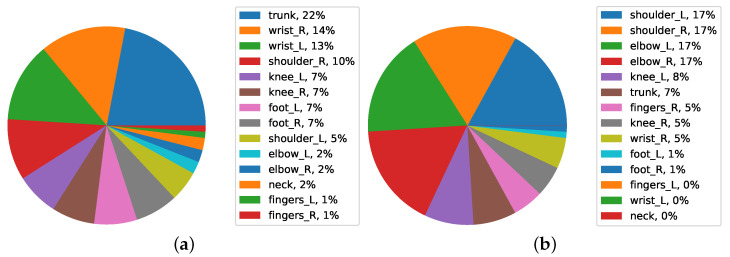
Average FWA of body parts in Paint (**a**) and (**b**) Quality Assurance data.

Figure 16 shows the pie chart for the paint category. Workers’ health protection was related to the trunk in the first level (22%) and the right wrist in the second level (14%). Considering the Quality Assurance data analysis using the plot, most of the health protection was related to the left elbow, right elbow, right shoulder, and left shoulder in the first level, with 17%. The analysis is especially useful for supporting a body part FWA loss model via determining how varied job demands influence changes in workers’ work ability, and in regulating which working conditions should be improved. The results are different categorizations of data, and the highest percentage is specialized in the trunk, while the second level is specialized in the right shoulder.

XAI methods are broadly used in many applications association with healthcare, especially pain modeling [52]. The utilization of AI in healthcare has reduced the burden on the biomedical system exclusively. For example, Hines et al. explored the issue of persistent pain experienced after receiving a total knee replacement. Using ML methods such as K-Nearest Neighbors (KNN), Support Vector Machine (SVM), and discriminate analysis were then introduced as a Lime technique to classify the varying levels of damage present by signal processing [53]. The recent research also shows that investment of AI in healthcare has increased tremendously in recent years, with predicting readmission risk among the frail being assisted by AI systems that detect and assess the health of patients instantly with feature analysis with SHAP [54].

Musculoskeletal symptoms vary with time and are not evaluated using a worker’s snapshot. Different research has been conducted with HCXAI in prevention and has proved that musculoskeletal symptoms are the most difficult to predict, among others [36]. There was little research that went on to discuss it through the history of medical appointments, especially in the automotive industry. However, we achieved certain progress in prognosis not only in the next medical appointment but also in the next body part severity, as discussed above in this paper. Incorporating XAI models can interpret the features that are explainable to cause work-related absenteeism and highlight their importance in prognosis.

## 5. Conclusions and Future Work

Work-related symptoms have a global influence on people’s well-being and quality of life and can be a financial burden for organizations by reducing productivity, increasing absenteeism, and promoting early retirement. Work-related musculoskeletal symptoms, in particular, represent a significant fraction of the total in all occupational contexts. In automotive and industrial settings where workers are exposed to work-related musculoskeletal disorders risk factors, OP are responsible for monitoring workers’ health protection profiles. Occupational technicians report in the OHPPs database to understand which exposure to occupational work-related musculoskeletal disorder risk factors should be ensured for a given worker. OHPPs databases describe the occupational physician states, and which exposure the physicians consider necessary to ensure the worker’s health protection in terms of their functional work ability. The application of HCXAI can support the decision making to go from worker’s Functional Work Ability to explanations by integrating explainability into medical (restriction) and support in two decision contexts: (1) prediction of next medical appointment (2) prediction of next body part(s) health protection. Although previous ML approaches (e.g., [36,53]) provided good predictions, their application in an actual clinical setting is limited because their predictions are difficult to interpret, and hence, not actionable. In this study, our target is the analysis of body parts that affected the worker’s functional work ability status. On the one hand, AI algorithms can help technical teams, occupational physicians, and ergonomists determine a worker’s workplace risk body part(s); these approaches can help prevent work-related musculoskeletal symptoms by identifying which processes are lacking in working condition improvement and which workplaces have a better match between the remaining functional work abilities. A sample of 7857 OHPPs based on Functional Work Ability textual reports of the Portuguese language in automotive industry factory was taken from 2019 to 2021. Machine learning-based Natural Language Processing methods were implemented to extract standardized information.

Explaining and integrating machine learning models into a technical team’s support systems is an easy task in and of itself because these models must be based on uncertain, imbalanced, heterogeneous, and noisy datasets [55,56], which contain a large number of features but are often insufficient to allow precise modeling of the complex systems of occupational profiles. The main contribution and objective of this research satisfied in: (1) by choosing the most pertinent metrics gathered in actual working situations, ML-based approaches can elicit key characteristics of OHPPs, (2) to compare ML-based improved worker’s health profile models to determine the best appropriate prognostic algorithms for real-environment, (3) since it is measured in terms of effectiveness, efficiency, satisfaction related to causal understanding, and transparency for industrial technical teams like ergonomists, the causality and explainability of these ML algorithms correspond to a human-understandable model. Therefore, the evaluation of OHPPs factors were developed in a reliable HCXAI system to promote a trustworthy HCXAI system. The most suitable regression models to predict the next medical appointment for the body regions were the models based on CatBoost regression with corresponding error evaluation, respectively. In parallel, the prediction of the next FWA body parts based on these two errors reported by CatBoost as the best regression model for most body parts. This information can help technical industrial teams understand potential risk factors for OHPPs and identify warning signs of the early stages of musculoskeletal disorders and how to cope with work-related absenteeism [57]. In the future, we will try to use other remaining XAI techniques [58,59,60] in our model for further improvement. Different XAI techniques will help us to make our model more explainable to others.

## Figures and Tables

**Table 1:** Two rows of final tabular structured data.

	Medical Restrictions	Neck	Trunk	${\mathrm{Shoulder}}_{L}$	${\mathrm{Shoulder}}_{R}$	${\mathrm{Elbow}}_{L}$	${\mathrm{Elbow}}_{R}$	${\mathrm{Wrist}}_{L}$	${\mathrm{Wrist}}_{R}$	${\mathrm{Fingers}}_{L}$	${\mathrm{Fingers}}_{R}$	${\mathrm{Knee}}_{L}$	${\mathrm{Knee}}_{R}$	${\mathrm{Foot}}_{L}$	${\mathrm{Foot}}_{R}$
1	Should not perform tasks that involve movements of the right elbow.			MN	MN		SN								
	Must not perform tasks that involve movements above both shoulders.														
2	Must not perform tasks that involve movements of the left wrist and left fingers.							MN		MN	MN				
	Must not perform tasks that require force with fingers of both hands.														

**Table 2:** Statistics of seniority in number of years based on different gender.

Gender	Size of the Study Population	Length of Employment			
		Max	Min	Mean	SD
Female	154	27	4	13.95	7.78
Male	490	29	4	18.62	8.33

**Table 3:** Scoring to each body region(s) injuries.

	Trunk	Shoulder_L	Shoulder_R	Finger_L	Finger_R	Wrist_R	Wrist_L
worker	1.66	0.5	0.5	0.5	0.5	0.8	0.8

**Table 4:** Descriptive of body parts variables.

	Count	Mean	std	Min	25%	50%	75%	Max
Elbow_L_	544	0.72	0.64	0.19	0.25	0.50	1.00	3.50
Shoulder_R_	651	0.74	0.61	0.19	0.28	0.50	1.00	3.50
Elbow_L_	186	1.05	0.76	0.19	0.00	0.00	0.00	3.50
Elbow_R_	241	1.09	0.83	0.19	0.50	0.75	1.58	3.50
Wrist_L_	231	1.16	0.84	0.25	0.50	1.00	1.58	3.60
Wrist_R_	237	1.09	0.95	0.25	0.50	0.75	1.50	5.33
Finger_L_	165	0.73	0.70	0.21	0.25	0.50	0.88	3.50
Finger_R_	185	0.76	0.72	0.21	0.25	0.50	1.00	3.50
Knee_L_	72	0.81	0.60	0.25	0.38	0.50	1.00	3.50
Knee_R_	94	1.18	0.88	0.25	0.50	1.00	1.50	4.00
Foot_L_	154	0.85	0.43	0.25	0.50	0.83	1.25	2.50
Foot_R_	154	0.85	0.43	0.25	0.50	0.83	1.25	2.50
Trunk	292	1.73	1.09	0.38	0.67	1.50	2.75	3.50
Neck	103	1.23	0.98	0.38	0.50	0.67	1.65	3.50

**Table 5:** ML regression models results.

Model	RMSLE	R2	CV_R2
Decision Tree	1.4516	0.6269	0.641 ± 0.02
Random Forest	1.2752	0.8197	0.8 ± 0.026
H_2_O Gradient Boosting	1.3487	0.5509	0.546 ± 0.033
Gradient Boosting	1.269	0.8251	0.797 ± 0.033
LGBM	1.2821	0.8159	0.771 ± 0.025
CatBoost	**1.2344**	**0.8497**	0.8 ± 0.028
XGBRegressor	1.2631	0.8344	0.794 ± 0.03

**Table 6:** Results of severity for the left shoulder.

Model	RMSLE	R2	MAE	CV-R2
Decision Tree	1.0235	0.6112	0.1115	0.459 ± 0.161
Random Forest	1.0025	0.8504	0.1175	0.709 ± 0.158
Gradient Boosting	1.0187	0.7523	0.1412	0.684 ± 0.163
LGBM	1.029	0.8735	0.1537	0.732 ± 0.114
CatBoost	0.9753	0.8555	0.1604	0.749 ± 0.144
XGBRegressor	1.0158	0.8179	0.1453	0.688 ± 0.157

**Table 7:** Results of severity for the right shoulder.

Model	RMSLE	R2	MAE	CV-R2
Decision Tree	1.0256	0.1255	0.0965	0.417 ± 0.119
Random Forest	0.997	0.6732	0.1968	0.66 ± 0.034
Gradient Boosting	0.98	0.5518	0.238	0.653 ± 0.042
LGBM	1.0311	0.7138	0.1929	0.716 ± 0.055
CatBoost	0.9433	0.7255	0.266	0.701 ± 0.073
XGBRegressor	0.975	0.5862	0.2405	0.648 ± 0.019

**Table 8:** Results of severity for the left elbow.

Model	RMSLE	R2	MAE	CV-R2
Decision Tree	0.6169	0.6735	0.0256	0.418 ± 0.121
Random Forest	0.6113	0.644	0.0397	0.697 ± 0.051
Gradient Boosting	0.6098	0.6555	0.0594	0.638 ± 0.003
LGBM	0.6225	0.6017	0.0923	0.694 ± 0.08
CatBoost	0.6102	0.0629	0.0629	0.706 ± 0.086
XGBRegressor	0.6067	0.6465	0.0659	0.621 ± 0.053

**Table 9:** Results of severity for the right elbow.

Model	RMSLE	R2	MAE	CV-R2
Decision Tree	0.7056	0.734	0.0562	0.685 ± 0.077
Random Forest	0.6861	0.8454	0.1092	0.815 ± 0.094
Gradient Boosting	0.6888	0.8268	0.1158	0.784 ± 0.076
LGBM	0.6817	0.7868	0.13	0.789 ± 0.062
CatBoost	0.6611	0.8223	0.1413	0.834 ± 0.07
XGBRegressor	0.6865	0.8318	0.1227	0.793 ± 0.094

**Table 10:** Results of severity for the trunk.

Model	RMSLE	R2	MAE	CV-R2
Decision Tree	0.9132	0.739	0.2602	0.844 ± 0.033
Random Forest	0.8948	0.8288	0.2925	0.89 ± 0.013
Gradient Boosting	0.8951	0.8306	0.307	0.853 ± 0.031
LGBM	0.8901	0.8684	0.1622	0.893 ± 0.015
CatBoost	0.8851	0.8443	0.3352	0.896 ± 0.017
XGBRegressor	0.9129	0.8416	0.3255	0.866 ± 0.03

## Data Availability

Not applicable.
